# 3D-bioprinting ready-to-implant anisotropic menisci recapitulate healthy meniscus phenotype and prevent secondary joint degeneration

**DOI:** 10.7150/thno.54864

**Published:** 2021-03-05

**Authors:** Ye Sun, Yuxin Zhang, Qiang Wu, Feng Gao, Yongzhong Wei, Yimin Ma, Wenbo Jiang, Kerong Dai

**Affiliations:** 1 Department of Orthopaedics, The First Affiliated Hospital of Nanjing Medical University, Jiangsu, 210029, China.; 2 Clinical and Translational Research Center for 3D Printing Technology, Shanghai Ninth People's Hospital, Shanghai Jiao Tong University School of Medicine, Shanghai, 200011, China.; 3 Shanghai Key Laboratory of Orthopaedic Implants, Department of Orthopaedic Surgery, Shanghai Ninth People's Hospital, Shanghai Jiao Tong University School of Medicine, Shanghai, 200011, China.; 4 Department of Rehabilitation Medicine, Shanghai Ninth People's Hospital, Shanghai Jiao Tong University School of Medicine, Shanghai, 200011, China.

**Keywords:** meniscus, 3d-print, bio-print, hydrogel, tissue engineering, chondrocyte, vascularization

## Abstract

**Objectives:** Disruption of anisotropic phenotypes of the meniscus would contribute to OA progression. Exploring phenotype changes of the anisotropic meniscus in joint degeneration would help understand the biologic interaction between the meniscus and OA, and further facilitate the therapeutic strategies of meniscus injury-related joint degeneration. Meanwhile, engineering biomimetic meniscal tissue mimicking the anisotropy of the healthy meniscus remains a challenge.

**Methods & Results:** Meniscal disruption of phenotype anisotropy (PBV growth, cellular phenotype and ECM depositions) was confirmed in OA patient samples. To recapitulate healthy meniscus phenotypes, 3D-bioprinted anisotropic TCM meniscus constructs with PBV growth and regional differential cell and ECM depositions were generated. Transplanted 3D-bioprinted meniscus into rabbit knees recapitulated phenotypes of native healthy meniscus and conferred long-term protection against secondary joint degeneration.

**Conclusion:** 3D-bioprinted TCM meniscus not only restored the anisotropy of native healthy meniscus with PBV infiltration and better shape retention, but better maintained joint function and prevented secondary joint degeneration, which provided a new strategy for the clinical treatment of meniscus injury-related joint degenerative diseases.

## Introduction

Traumatic injuries and degeneration of meniscus are important risk factors for osteoarthritis (OA) and joint dysfunction 1,2. In clinical practice, nearly half patients with meniscus rupture develop OA over time 3. Meniscus is a crescent-shaped cartilaginous tissue between the distal femoral and proximal tibial condyles, providing structural congruence and absorbing mechanical forces 4. Meniscus degeneration or meniscectomy exposes knee joint cartilage to excessive stress, leading to secondary arthritis in the long term 4. Healthy meniscus demonstrates complex anisotropic phenotype in the outer and inner regions. In particular, blood vessels at the peripheral region provide nutrient supply and help maintain the regional cellular phenotype while fibroblast-like chondrocytes (FC) exist within an extracellular matrix (ECM) comprising mainly type I collagen, contributing to its resistance against the tensile loads 5. Meanwhile, articular chondrocyte-like cells (AC) are embedded in the inner region within an ECM rich in type II collagen (COL- II) and glycosaminoglycans (GAGs), enabling resistance against inner compression of the joint 6. However, little is known about how the anisotropic nature of the meniscus would contribute to OA progression. Exploring phenotype changes in the anisotropic meniscus in joint degeneration would help understand the molecular interaction between meniscus degeneration and OA, and further facilitate the therapeutic strategies of meniscus injury-related joint degeneration. Moreover, engineering biomimetic meniscal tissue mimicking the anisotropy of the healthy meniscus remains a challenge 7,8. The engineered meniscal tissue should not only restore the anisotropy of cells and matrix, but also show the growth of peripheral blood vessels (PBV) for the supply of nutrients, in the long run to withstand stress and prevent OA progression 9.

Three-dimensional (3D)-printed biomaterial constructs have been tried to generate different human tissues, including meniscus 10-13.

Bioprinterscan deliver cells within hydrogels, biomaterials and other molecules to engineer 3D living tissue 14-16. With the process optimization, upgrade of printing software, and the development of coordinated multi-nozzle printing systems, the construction of bionic tissues and organs with different structural, heterogeneous components, and diverse cell types has become possible 17-19.

Here, we report a 3D-bioprinted anisotropic meniscus scaffolds with PBV growth, regional differential cell and ECM depositions. Transplanted 3D-bioprinted meniscus into rabbit joints recapitulated phenotypes of native healthy meniscus and conferred long-term protection against secondary joint degeneration.

## Results and Discussion

### Regional anisotropic meniscus phenotype was disrupted with meniscus degeneration in OA development

**Figure 1:**
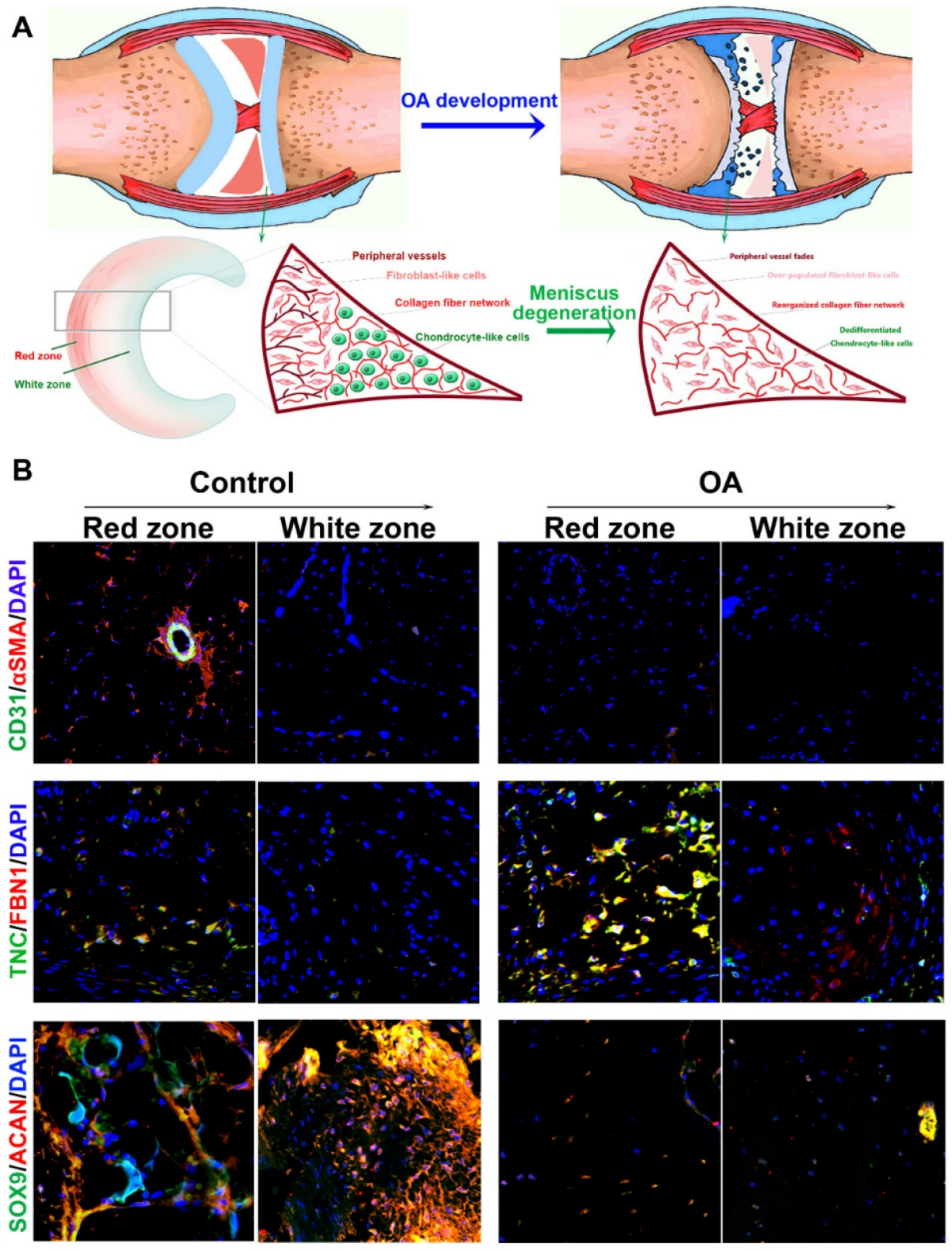
** Disruption of meniscal phenotype anisotropy in OA development. A.** Schematic illustration of meniscal disruption of phenotype anisotropy (PBV growth, cellular phenotype and ECM depositions) in OA development. **B.** Evaluation of altered anisotropic meniscus phenotype in OA development with immunofluorescence. the regional meniscus phenotypes were compared in OA patients and healthy controls. In the healthy meniscus, PBV growth was demonstrated in the outer red zone with positive immunostaining of blood vessel markers CD31 (green) and αSMA (red). Moreover, chondrocytes in OA meniscus demonstrated severe fibroblastic changes (TNC: green; FBN1: red) and disappearing of articular cartilage phenotypes (SOX9: green; ACAN: red) respectively.

**Figure 2:**
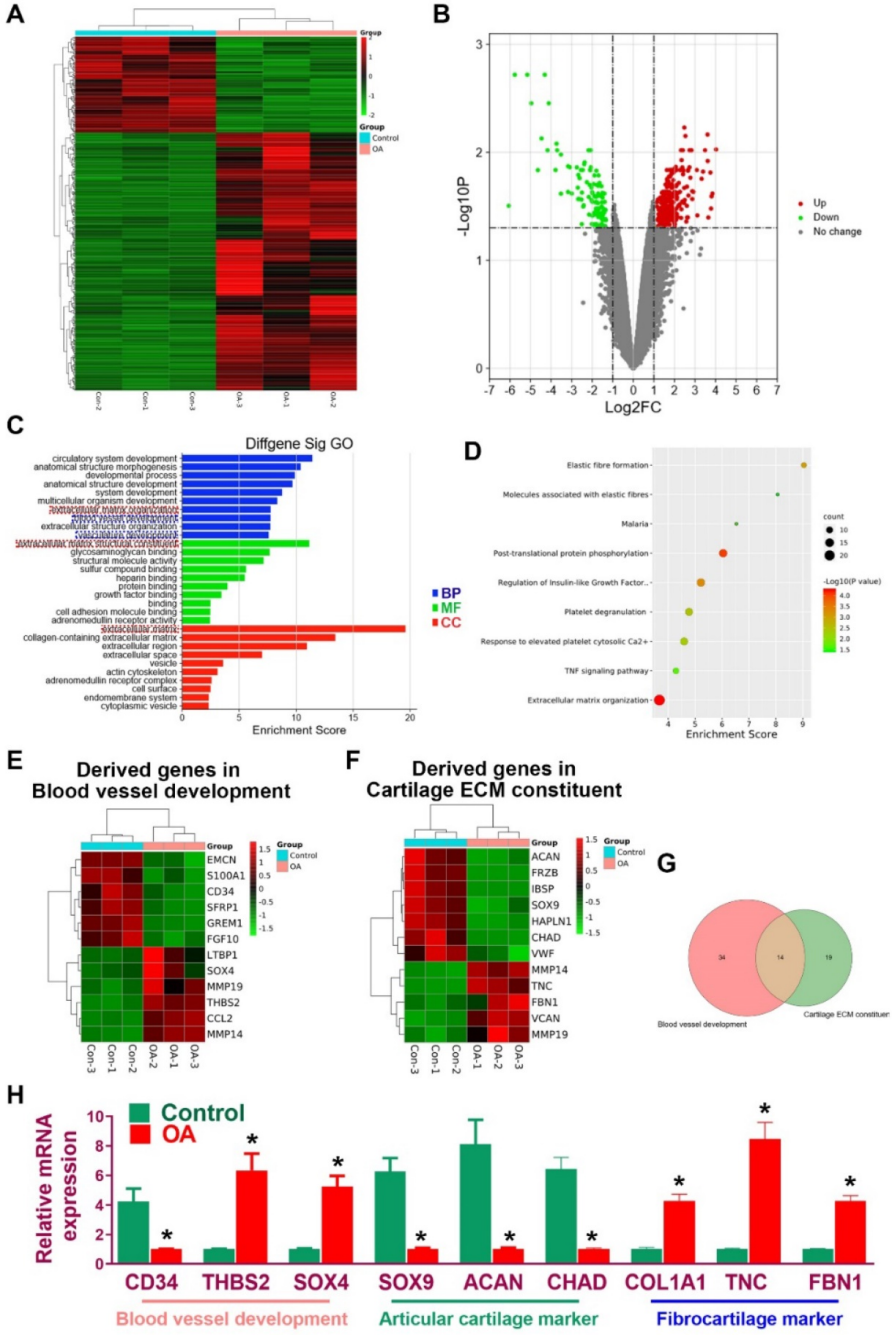
** Disruption of gene expression profiles in OA meniscus. A.** Heatmap of clustering dysregulated mRNA expression profiles with microarray in OA meniscus compared to healthy control. **B.** Volcano plot of mRNA expression profiles of meniscus in OA development. **C.** All differentially expressed genes (DEG with fold change >2 or <0.5, p value <0.01) were subjected to gene ontology (GO) analysis. Downregulated GO terms of altered meniscal phenotype anisotropy were related to extracellular matrix organization, vasculature development, extracellular matrix structure constituent BP: biological processes, MF: molecular function, CC: cellular component. **D.** Significantly enriched pathways for OA meniscus in Kyoto Encyclopedia of Genes and Genomes (KEGG) pathways. **E-G.** Enriched genes for blood vessel development (n=34) and cartilage ECM constituent (n=19) were derived from the mRNA microarray results. **E)** Various blood vessel markers (e.g. EMCN, CD34, GREM1) and** F)** typical cartilage markers (e.g. ACAN, FRZB, SOX9, HAPLN1 and CHAD) were downregulated in OA-derived meniscus tissues. Meanwhile, fibroblastic markers (e.g. TNC, FBN1, VCAN and MMP14) were significantly upregulated in OA-derived meniscal tissues in OA patients. **H.** Dysregulation of blood vessel markers and cartilage markers were further validated with qRT-PCR, confirming disruption of meniscus phenotype anisotropy concerning blood vessel development and regional differential chondrocyte phenotypes. Data are presented as averages ± SD and were analyzed by two-way analysis of variance (ANOVA) with Tukey's test. *P< 0.05 between OA group and the control group.

In the present study, we hypothesized that the meniscal disruption of phenotype anisotropy (PBV growth, cellular phenotype and ECM depositions) might contribute to OA development (Figure 1A). To explore the biological interaction between anisotropic meniscus phenotype and OA development, the regional meniscus phenotypes were evaluated and compared in OA patients and healthy controls. In the healthy meniscus, PBV growth was demonstrated in the outer red zone with positive immunostaining of blood vessel markers CD31 and αSMA (Figure 1B). Moreover, chondrocytes in the red zone and white zone demonstrated fibrocartilage (Fibrocartilage marker: TNC & FBN) and articular cartilage (articular cartilage marker: SOX9 & ACAN) phenotypes respectively (Figure 1B). In comparison, meniscus phenotype anisotropy was disrupted in OA, showing severe fibroblastic changes, PBV disappearing in the red zone and dedifferentiated chondrocyte phenotypes in both zones (Figure 1B). To further decipher the molecular changes underlying the phenotype anisotropy disruption, dysregulated mRNAs were identified in meniscal tissues (mixed samples from both red and white zones) from OA patients and controls (Figure 2A-B). All these genes were subjected to gene ontology (GO) analysis (Figure 2C). Downregulated gene GO terms concerning chondrogenesis and OA development for biological processes, molecular function, and cellular component were related to ECM organization, vasculature development, ECM structure constituent (Figure 2C). Moreover, elastic fiber formation, TNF signaling pathway, and ECM organization were significantly enriched for OA-derived meniscus tissues in Kyoto Encyclopedia of Genes and Genomes (KEGG) pathways (Figure 2D). Enriched genes for blood vessel development (n=34) and cartilage ECM constituent (n=19) were derived from the mRNA microarray results (Figure 2E-G). Various blood vessel markers (e.g. EMCN, CD34, GREM1) and typical cartilage markers (e.g. ACAN, FRZB, SOX9, HAPLN1, and CHAD) were downregulated in OA-derived meniscus tissues. Meanwhile, fibroblastic markers (e.g. TNC, FBN1, VCAN, and MMP14) were significantly upregulated in OA-derived meniscal tissues in OA patients. Dysregulation of these markers were further validated with qRT-PCR (Figure 2H), confirming the disruption of meniscus phenotype anisotropy concerning blood vessel development and regional differential chondrocyte phenotypes.

### Generating anisotropic menisci constructs to recapitulate healthy meniscus phenotype with 3D-bioprinting

**Figure 3:**
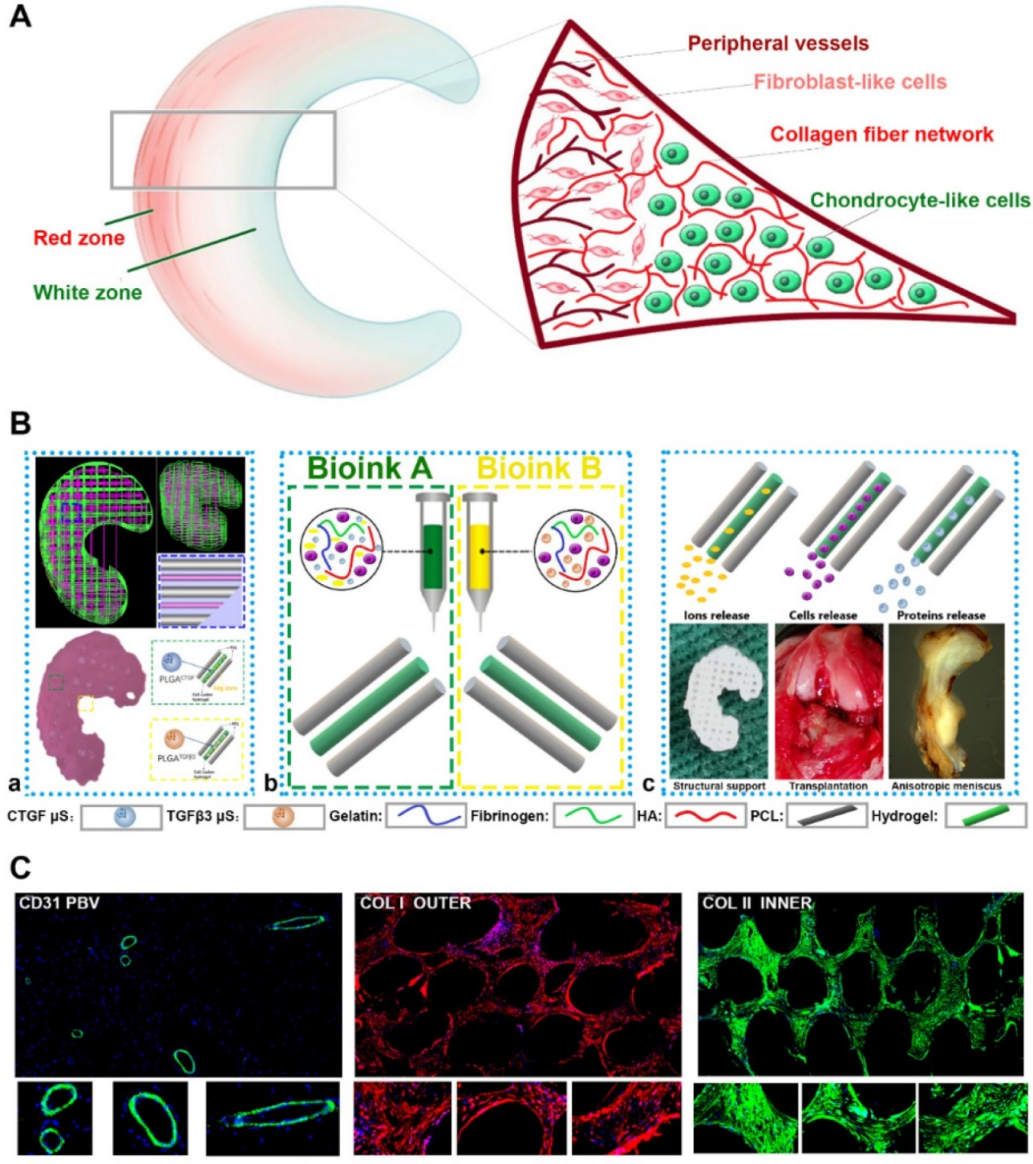
** Schematic presentation of the study design and scaffold construction. A.** Schematic main cell types in the red zone and white zone of healthy meniscus. The inner 2/3 region is populated with oval-shaped articular chondrocytes situating in well-formed lacunae with collagen II-rich ECM, while the outer 1/3 region (red zone), nourished by its PBV for vascular supply, is mainly populated by spindle-shaped fibroblast-like chondrocytes and consists predominately of collagen type I. **B.** To recapitulate the phenotype anisotropy of healthy meniscus, 3D-bioprinted biomimetic TCM meniscus constructs were created. **a)** A CAD model was used to design the 3D-bioprinted rabbit meniscus. PCL scaffolding structure and bioinks were alternately printed to offer biomechanical support and phenotype anisotropy. Different growth factors were incorporated for anisotropic chondrogenic induction (CTGF for the outer region, TGFβ3 for the inner region) and angiogenesis (magnesium ion for the outer region) for PBV induction in the TCM meniscus.** b)** Bioink A was used in outer 1/3 region for PBV induction and fibrocartilage differentiation while Bioink B was used in inner 2/3 region for differentiation into articular cartilage.** c)** TCM meniscus construct provides structural support and region-specific release of magnesium ions, SMSCs and proteins for PBV induction, fibrocartilage and articular cartilage generation when transplanted in rabbits. Different components in the diagram were depicted in the lower panel. **C.** Phenotype anisotropy of the transplanted TCM meniscus was demonstrated with immunofluorescence for PBV induction (CD31 staining: green), fibrocartilage (COL I staining: red) and articular cartilage (COL II staining: green) generation. Magnified immunofluorescence images were listed in the lower panel.

**Figure 4:**
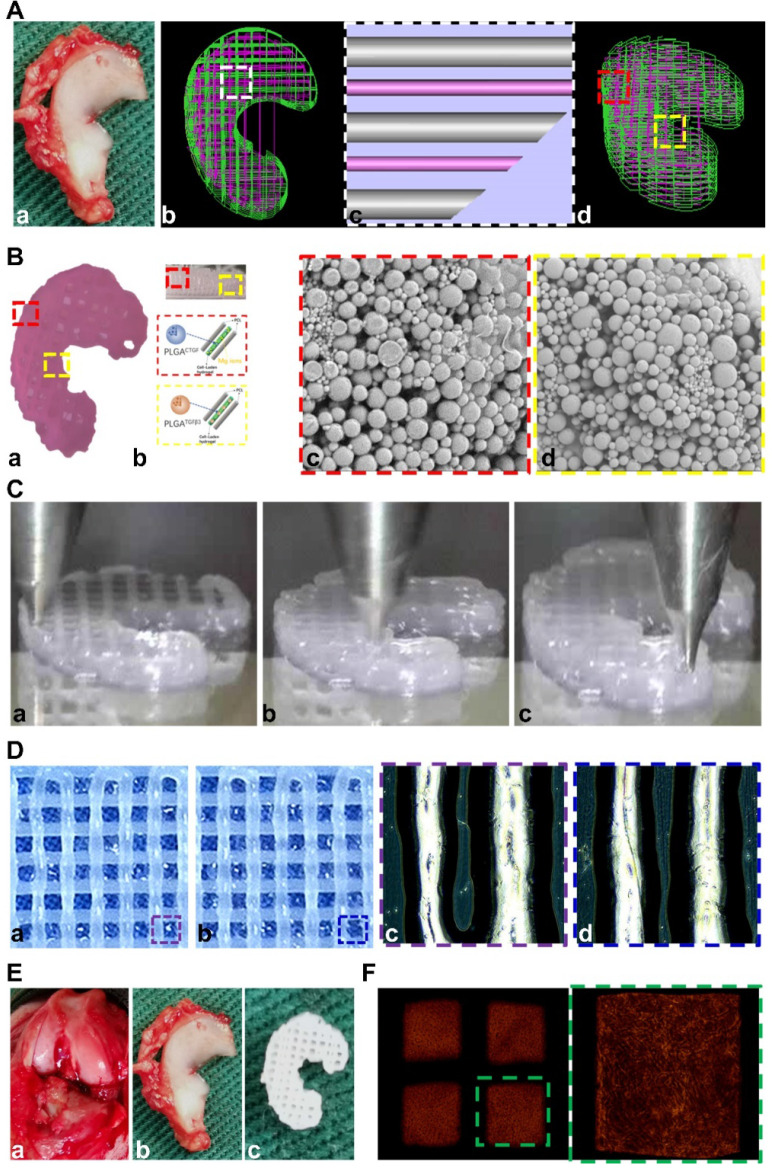
** Design and construction of 3d-bioprinted TCM meniscus scaffold. A. a)** The structure of native rabbit meniscus and** b-d)** a 3D CAD model (**b**: top view; **d**: side view) was developed from image data of rabbit meniscus to generate **c)** a visualized motion program for printing. **c)** A computer-generated 3D meniscus model can be converted to a motion program that operates and guides the dispensing nozzles to take defined paths for delivery of cell-laden hydrogel (purple) and PCL (silver). **B. a)** Gross appearance of the 3D-bioprinted TCM meniscus.** b)** MSC cell-laden hydrogel encapsulating PLGA microparticles carrying TGFβ3 (yellow box) or CTGF with Mg^2+^ (red box) in different regions were bio-printed into the microchannels (300 µm) between PCL fibers with bioinks from different syringes. **c-d)** SEM images of both PLGA µS (**c:** CTGF; **d:** TGFβ3) demonstrated a less than 2 µm diameter. **C.** Printing process of biomimetic TCM meniscus. (**a:** outer region; **b:** inner region; **c:** the final product) **D.** Printed scaffold showed **a-b)** delicate and orderly hydrogel-PCL alignment under light microscope. **c-d)** Multi-factor encapsulated MSC cell-laden hydrogel (**c**: CTGF & Mg^2+^; **d**: TGFβ3) showed nice printability. **E. a)** Transplantation of TCM meniscus showed good resemblance to **b)** native rabbit meniscus for **c)** the TCM meniscus. **F.** Delicate and orderly hydrogel-PCL alignment under light microscope with well-distributed abundant cells encapsulated in the crosslinked hydrogel (right panel: magnified image of the green box in the left panel).

Anisotropic menisci constructs were fabricated with the OPUS bioprinter to recapitulate the phenotype anisotropy of healthy meniscus in rabbits (Figure 3-4). Main cell type in the inner and middle part of the meniscus (white zone: avascular region) has been termed the articular chondrocytes with round or oval shape, situating in well-formed lacunae with collagen II-rich ECM. The outer 1/3 region (red zone: vascular region) of the meniscus, nourished by its PBV for vascular supply, is mainly populated by fibroblast-like cells with spindle cell form and consists predominately of collagen type I (Figure 3A). To recapitulate the phenotype anisotropy of healthy meniscus, biomimetic meniscus constructs for meniscus transplantation in rabbits were created by incorporating different growth factor release for chondrogenic induction (CTGF for the outer region, TGFβ3 for the inner region) and magnesium release for angiogenesis (for the outer region) in the TCM group (Figure 3B-C). A program-generated 3D meniscus model was used to generate a motion program, guiding the dispensing nozzles to take specific defined paths for delivery of cell-laden hydrogel and materials (Figure 4A). Briefly, PCL was molten to fabricate scaffolding structure for the meniscus while MSC-laden hydrogel encapsulating PLGA microspheres carrying TGFβ3 or CTGF & magnesium ions in different regions was deposited in the microchannels from different syringes (Figure 3B; Figure 4A-B). During plotting, the needle diameter, layer thickness and speed for PCL printing were kept constant at 200 μm, 200 μm, and 180 mm/min, respectively. The fiber spacing was kept constant at 300 μm according to our previous research, which would provide better mechanical support for the scaffold, allow a suitable cell density, and facilitate the uniform distribution and contact of cells, thereby promoting cell viability and proliferation 20,21. PLGA microspheres (μS) was used to deliver TGFβ3 and CTGF in the MSC-laden hydrogels. TGFβ3 was delivered in the inner 2/3 region for articular cartilage regeneration in bioink A, while CTGF and magnesium ions were co-delivered in bioink B in the outer 1/3 region for induction of fibro-chondrocyte phenotype and PBV ingrowth (Figure 3A-B). MSC-laden hydrogel with encapsulated PLGA microspheres showed nice printability as demonstrated in Figure 4C-D, and scanning electron microscope (SEM) images of both PLGA µS indicated a less than 2 µm diameter (Figure 4B). Printed scaffold presented delicate and orderly hydrogel-PCL alignment and good resemblance to the native rabbit meniscus during transplantation (Figure 4D-F). Further studies *in vitro* and transplantation *in vivo* were conducted to evaluate the phenotype anisotropy of generated TCM menisci scaffold (Figure 3C; Figure 4E-F).

**Figure 5:**
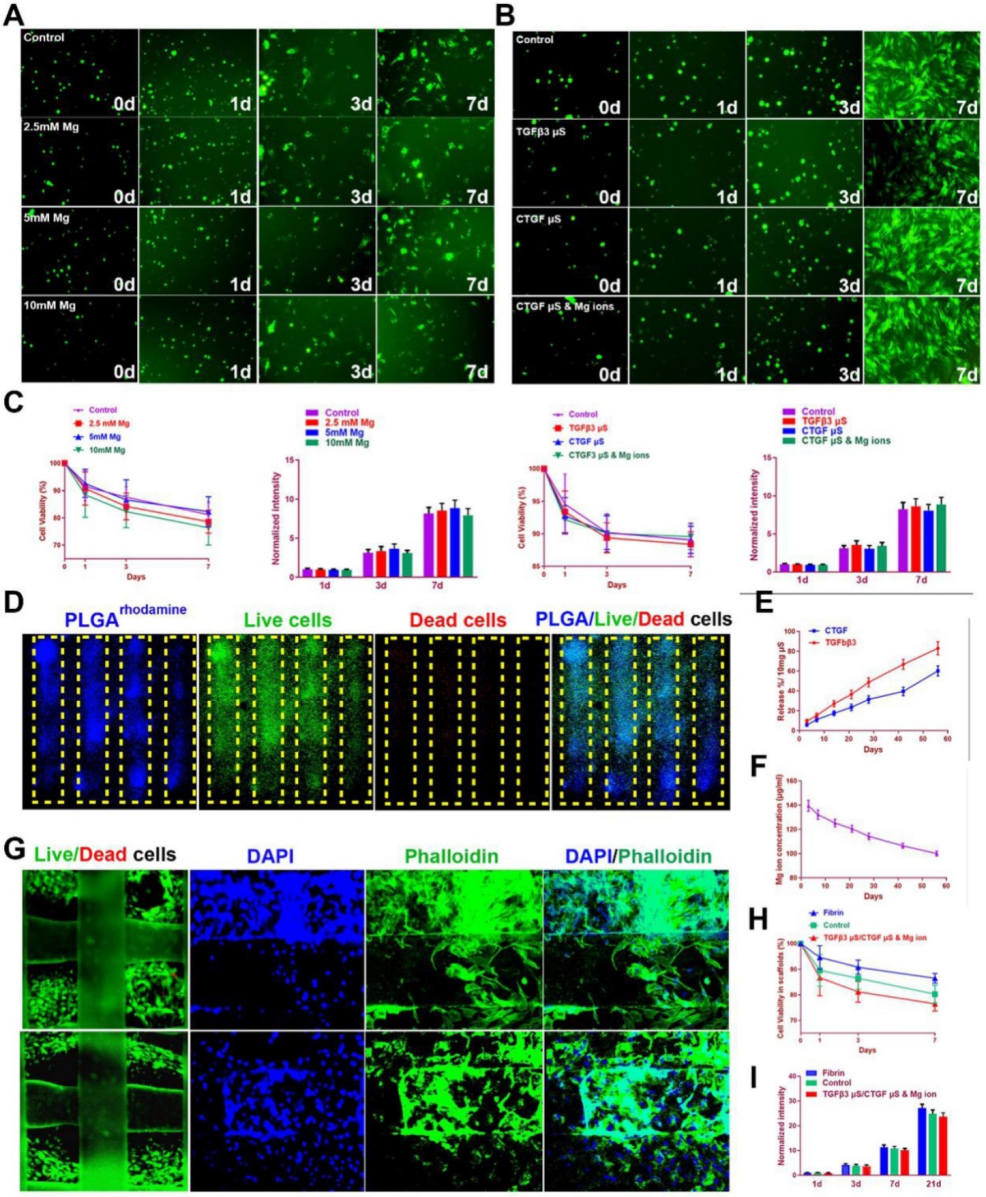
** SMSC viability and proliferation in the composite hydrogel and TCM scaffolds. A-B.** Cells (green by calcein dye) observed under fluorescent microscope in the composite hydrogel with different conditioned supplementations (**A:** 0-10 mM Mg^2+^; **B:** TGFβ3 µS, CTGF µS, and CTGF µS & 5 mM Mg^2+^) compared to cells in the control group over 7 days.** C.** Cell viability (1^st^ and 3^rd^ panels) by CCK-8 and cell proliferation (2^nd^ and 4^th^ panels) by Alamar blue assay kit in the composite hydrogel with different conditioned supplementations compared to the control group over 7 days**. D.** Microsphere distribution in SMSC-laden hydrogel in the constructed scaffolds. Fluorophore-conjugated rhodamine (blue) was encapsulated into PLGA µS and delivered to the hydrogel to validate µS distribution in cell-laden hydrogel. At day 7, PLGA^rhodamine^ µS showed well-proportioned distribution as well as minimal cell toxicity (by live/dead assay) in the hydrogel printed between the PCL fibers under confocal microscope (green for live cells, red for dead cells). The yellow frame indicates the microchannels between PCL scaffolding structure in the scaffold. **E.** Microsphere release of CTGF and TGFβ3 in SMSC-laden hydrogel of the meniscus construct using enzyme-linked immunosorbent assay (ELISA) kits over 60 days* in vitro*. **F.** Magnesium concentration was measured with Magnesium Assay Kit over 60 days* in vitro*. **G.** Immunostaining of cytoskeleton (blue for DAPI and green for Phalloidin) showed cell spreading in the hydrogel (upper panel: outer region; lower panel: inner region) and three-dimensional anchoring to the PCL fibers. **H.** Survival of SMSCs was examined over 21 day after printing with Live/dead cell assays compared to cells in the control scaffold or fibrin. **I.** Cell proliferation in the TCM constructs were examined with Alamar blue assay kit and compared to cells cultured in fibrin for 21 days after printing.

To test the effects of magnesium ions, CTGF, and TGFβ3 µS on SMSC viability and proliferation, SMSCs were cultured in the composite hydrogel for 7 days (Figure 5A). Similar effects on viability and proliferation of SMSCs were identified among different groups with different Mg^2+^ concentrations or PLGA µs through seven days in the hydrogel (Figure 5A-C). To validate microsphere distribution in the hydrogel, fluorophore-conjugated rhodamine was encapsulated into PLGA µS and delivered to the hydrogel, demonstrating well-proportioned distribution in the meniscus scaffold as well as minimal cell toxicity in the hydrogel under confocal microscope (Figure 5D). Released CTGF, TGFβ3, and magnesium ion concentration was measured using enzyme-linked immunosorbent assay (ELISA) kits. Relatively rapid TGFβ3 release was shown with slower release of CTGF over 60 days *in vitro* (Figure 5E). Meanwhile, concentration of magnesium ions were sustained over 100 µg/ml during this period (Figure 5F). Cell viability was further shown in the post-printed scaffolds (Figure 5G-I). Immunostaining of cytoskeleton showed cellular spreading and good anchoring to the PCL fibers (Figure 5G) 22,23. Live/dead cell assays indicated ≥95% cell viability post-printing (Figure 2F), which was maintained above 85% with no significant difference compared to cells in fibrin or control (without chemical stimulation) through 7 days (Figure 5H). Meanwhile, proliferation was neither affected in the scaffolds after 21 days post printing (Figure 5I). These data demonstrated that the meniscus scaffolds, which recapitulate the phenotype anisotropy of healthy meniscus, could sustain cell viability and provide a suitable microenvironment for SMSC survival, spreading and differentiation to region-specific chondrocytes *in vitro*.

### Magnesium ions promote tube formation and HUVEC migration into the bioprinted meniscus scaffold *in vitro*

**Figure 6:**
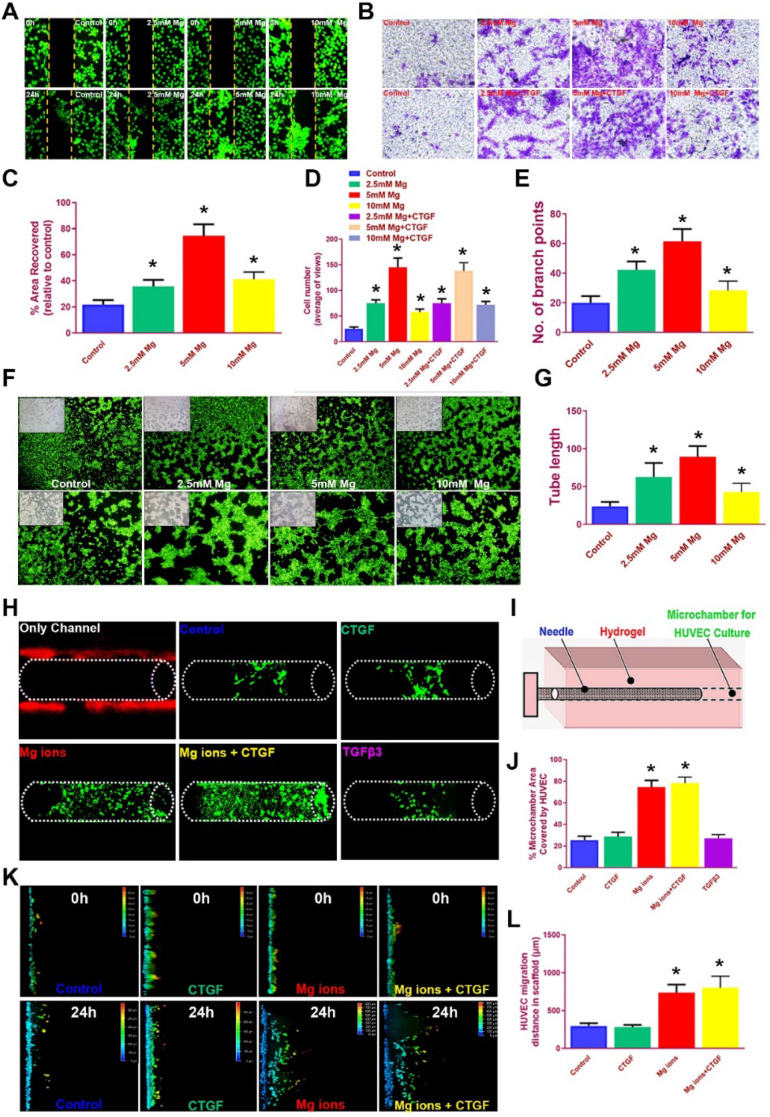
** Magnesium promoted migration and blood vessel formation by HUVECs. A.** Scratch assay to demonstrate the Mg^2+^-induced wound healing capability of HUVECs in hydrogel over 24 hours. Different concentrations of Mg^2+^ (0-10 mM) were used. HUVECs were stained with calcein dye.** B.** The effects of multi-factor treatment (0-10 mM Mg^2+^ w/wo CTGF) on HUVECs migration observed under light microscopy in different treatment groups with transwell assay. **C.** Covered areas in the scratched area of the hydrogel over 24 hours in the scratch test for both groups (n=6 for each). **D.** Number of migrated HUVECs in different treatment groups (n=6 for each) in transwell assay. **E-G.** Angiogenesis potential by HUVECs treated with different concentrations of Mg^2+^ in the tube formation assay. **E)** Number of branch points and **G)** quantified tube length in **F)** the tube formation assay. HUVECs were stained with calcein dye.** H-J.** Migration of HUVECs in the bioprinted hydrogel with different stimuli combinations. **H)** HUVECs were seeded in the hydrogel and **I)** a microchamber was punched out with a needle. **J)** Magnesium (w/wo CTGF) significantly elevated the percent of recovered areas by the migrated HUVECs within.** K-L.** Migration assay of HUVECs over 24 hours in the PCL scaffolds under confocal microscopy in Mg^2+^-treated (w/wo CTGF) groups and the control group. The total migration distance in height axis of the confocal microscopy for examination was 1.5 mm. **F.** Comparison of the migration distance for HUVECs in the PCL scaffolds over 24 hours (n=6 for each). Data are presented as averages ± SD and were analyzed by two-way analysis of variance (ANOVA) with Tukey's test. *P< 0.05 between other treatment groups and the control group.

It is acknowledged the HUVECs recruitment and migration to the outer region of the meniscus scaffolds *in vivo* is vital for PBV formation to offer nutrient supply, leading to better generated anisotropy. We examined the effects of magnesium on HUVEC migration in different groups with scatch and transwell assay (Figure 6A-D). Magnesium ion supplementation significantly enhanced the migration of hydrogel-embedded HUVEC into the scratch area, with the most covered areas by 5 mM Mg^2+^ over 24 hours (Figure 6A, 6C). Besides, number of migrated HUVECs in the groups with magnesium ions (with or without CTGF) were also significantly greater compared to the control group in transwell analysis (Figure 6B, 6D). Meanwhile, magnesium supplementation significantly enhanced the angiogenesis potential by HUVECs in the tube formation assay (Figure 6E-G). Furthermore, we used bioprinted hydrogels and PCL scaffolds to validate the migration of HUVEC by magnesium ions (Figure 6H-L). HUVECs were seeded into the bioprinted composite hydrogel, and a microchamber was punched out with a needle in the hydrogel with different stimuli combinations. Magnesium supplementation (with or without CTGF) significantly elevated the percent of recovered areas by the migrated HUVECs within (Figure 6H-J). Meanwhile, we placed PCL scaffolds atop the monolayer-cultured HUVEC incubated for 2 weeks *in vitro*. Consistently, scaffolds with magnesium ions demonstrated the greatest migration distance for HUVECs in the scaffolds on confocal microscope (Figure 6K-L). Therefore, we assume the chemical stimuli by magnesium ions/CTGF µS in the outer region of anisotropic meniscus scaffold could significantly enhance endogenous HUVEC migration toward the scaffold in the outer region, providing a good prospect for PBV ingrowth into the outer region of the scaffold (Figure 3C).

### Spatiotemporally released factors in 3D-bioprinted TCM meniscus scaffold recapitulate anisotropic cartilaginous matrix phenotype of healthy meniscus *in vitro*

**Figure 7:**
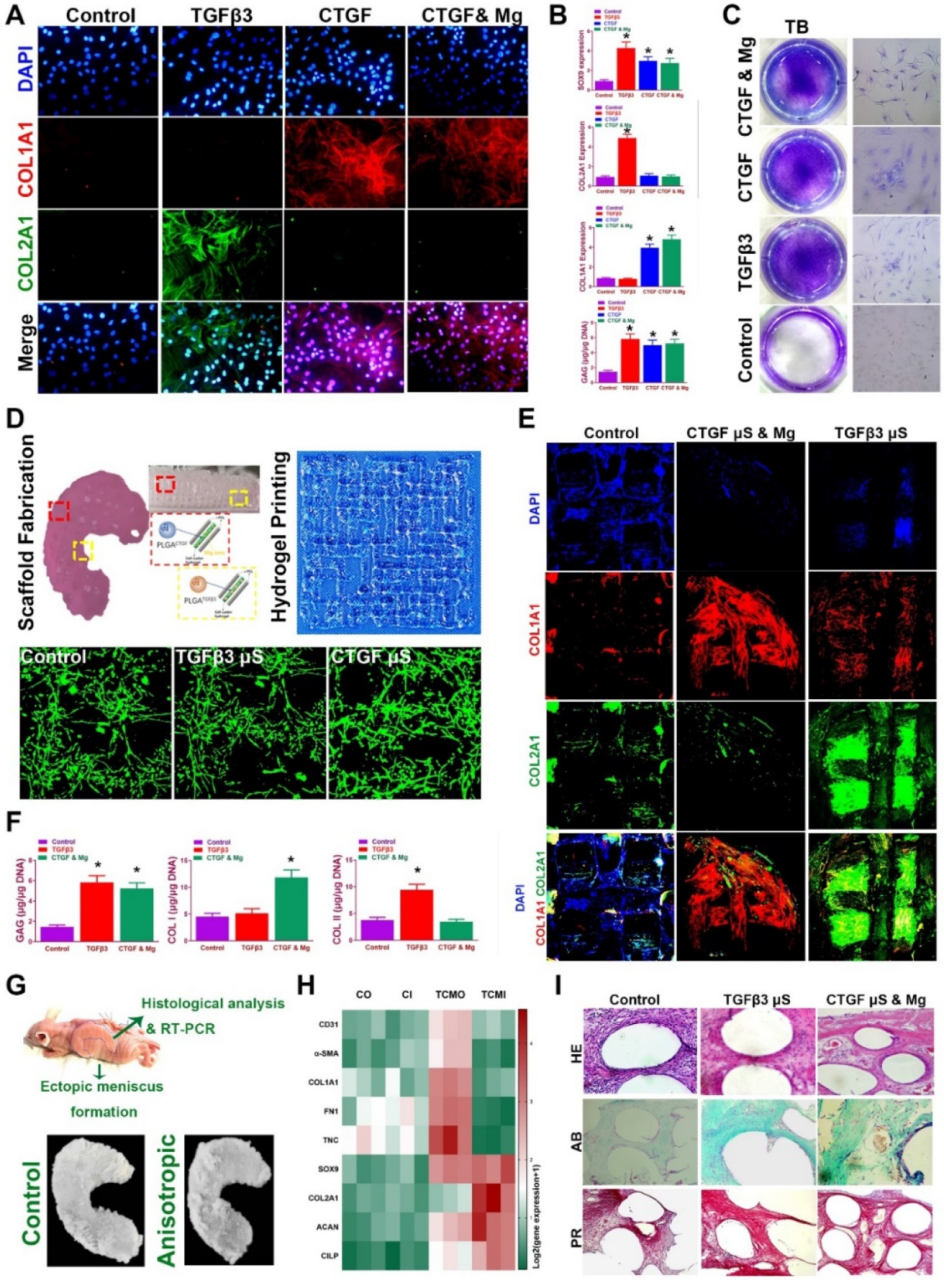
** Spatiotemporal delivery of conjugated factors induced region-specific chondrocyte phenotypes resembling healthy meniscus. A.** Immunofluorescence analysis of chondrogenic differentiation of SMSCs in the composite hydrogel with CTGF, TGFβ3, and CTGF with Mg^2+^ versus the control group (blue, nucleus; red, COL I; green, COL II) **B.** Gene expression of the chondrogenic markers SOX9, Col I, Col II and quantification of GAG depositions in different treatment groups in (A). **C.** Toluidine blue staining for GAG depositions in (A). **D.** Fabricated TCM meniscus (top left) was incubated *in vitro* for 12 weeks. Addition of PLGA µS did not alter the printability of the composite hydrogel and its orderly alignment in printing (top right). Cellular interaction and viability (lower panel) was observed under microscope for different µS-conjugated hydrogel compared to that in the control group. **E.** Immunofluorescence observation of regionally specific differentiation of MSCs in TCM meniscus (outer: CTGF & Mg2+; inner: TGFβ3) versus control meniscus (blue, nucleus; red, COL I; green, COL II). **F.** Quantification of Collagen I, collagen II and GAG depositions in different regions of the TCM meniscus compared to the control meniscus. **G.** TCM meniscus was transplanted subcutaneously for ectopic chondrogenesis in nude mice, showing cartilaginous matrix formation (lower panel) throughout the whole TCM scaffolds after 3 months. **H.** Expression levels of blood vessel markers, fibroblastic markers and cartilage markers were compared between TCM and control meniscus with qRT-PCR (CO: control outer; CI: control inner; TCMO: TCM outer; TCMI: TCM inner). **I.** Cartilaginous matrix formation and blood vessel ingrowth in different regions of TCM meniscus with HE and picrosirius red staining. All data are presented as means ± SD (n = 6) and were analyzed by two-way analysis of variance (ANOVA) with Tukey's test. *P< 0.05 between other treatment groups and the control group.

Before transplantation *in vivo* of the meniscus construct, we validated whether spatiotemporal delivery of conjugated factors could induce region-specific chondrocyte phenotypes that resemble the healthy meniscus. Chondrogenesis was defined with immunostaining and qRT-PCR for different combinations at two weeks *in vitro* (Figure 7A-C). CTGF (or with Mg2+) and TGFβ3 induced *COL1A1* and *COL2A1* expression respectively* in vitro* (Figure 7A-B), and all elevated SOX9 expression and GAG deposition compared to control group (Figure 7B-C). Meanwhile, CTGF (or with Mg^2+^) and TGFβ3 yielded a cartilaginous ECM formation that stained positive for toluidine blue, indicative of the formed, cartilage-like matrix (Figure 7C). Cartilaginous matrix phenotypes were further examined in the 3d-bioprinted scaffolds after 12-week culture* in vitro* (Figure 7D-F)*.* Scaffold was fabricated with healthy meniscus structure and delicate alignment of microsphere-conjugated SMSC-laden hydrogel printing were separately conducted (Figure 7D, upper panel). Different µS supplementation did not impede the printability of the hydrogel or its orderly alignment during printing (Figure 7D). No significant change in appearance or cell viability was observed for µS-conjugated hydrogel compared to the control group (Figure 7D, lower panel). Printed SMSC-laden hydrogel demonstrated regular-shaped cell alignment along the longitudinal direction of the printing paths, forming a reticular network by cell-cell interaction (Figure 7D, lower panel). In the printed scaffolds, spatiotemporally released CTGF with Mg^2+^ and TGFβ3 also deposited proteoglycan-rich ECM in the whole scaffold (Figure 7E-F), and induced significantly higher COL1A1 deposition in the outer and greater COL2A1 deposition in the inner region respectively (Figure 7E-F), recapitulating the anisotropic cartilaginous phenotype of native healthy meniscus.

To further determine the chondrogenic properties of the generated TCM meniscus scaffold, the scaffolds were transplanted subcutaneously for ectopic chondrogenesis in nude mice (Figure 7G-I). Cartilaginous tissues generated *in vivo* confirmed better chondrogenic lineage committed by the SMSCs in the TCM scaffolds with qRT-PCR, demonstrating anisotropic chondrogenic phenotypes with differential expressions of AC and FC markers in the outer region (TCMO) and inner region (TCMI) of the TCM scaffold (Figure 7H). Histological examination of the transplantation sites was conducted (Figure 7I). Compared to control, TCM scaffold deposited rich GAG overall indicated by ingrained metachromatic staining with alcian blue (AB). Meanwhile, abundant collagen I was observed with minor blood vessel in-growth in the outer region of the TCM scaffold with HE and picrosirius red staining (Figure 7I). These results suggested that TCM meniscus scaffold could generate anisotropic ectopic cartilaginous tissues with outer-region PBV ingrowth *in vivo*, indicating its prospective potential in anisotropic healthy meniscus regeneration when transplanted into the knee joint.

### TCM meniscus transplantation *in situ* restored phenotype anisotropy of healthy meniscus and prevented secondary OA development* in vivo*

**Figure 8:**
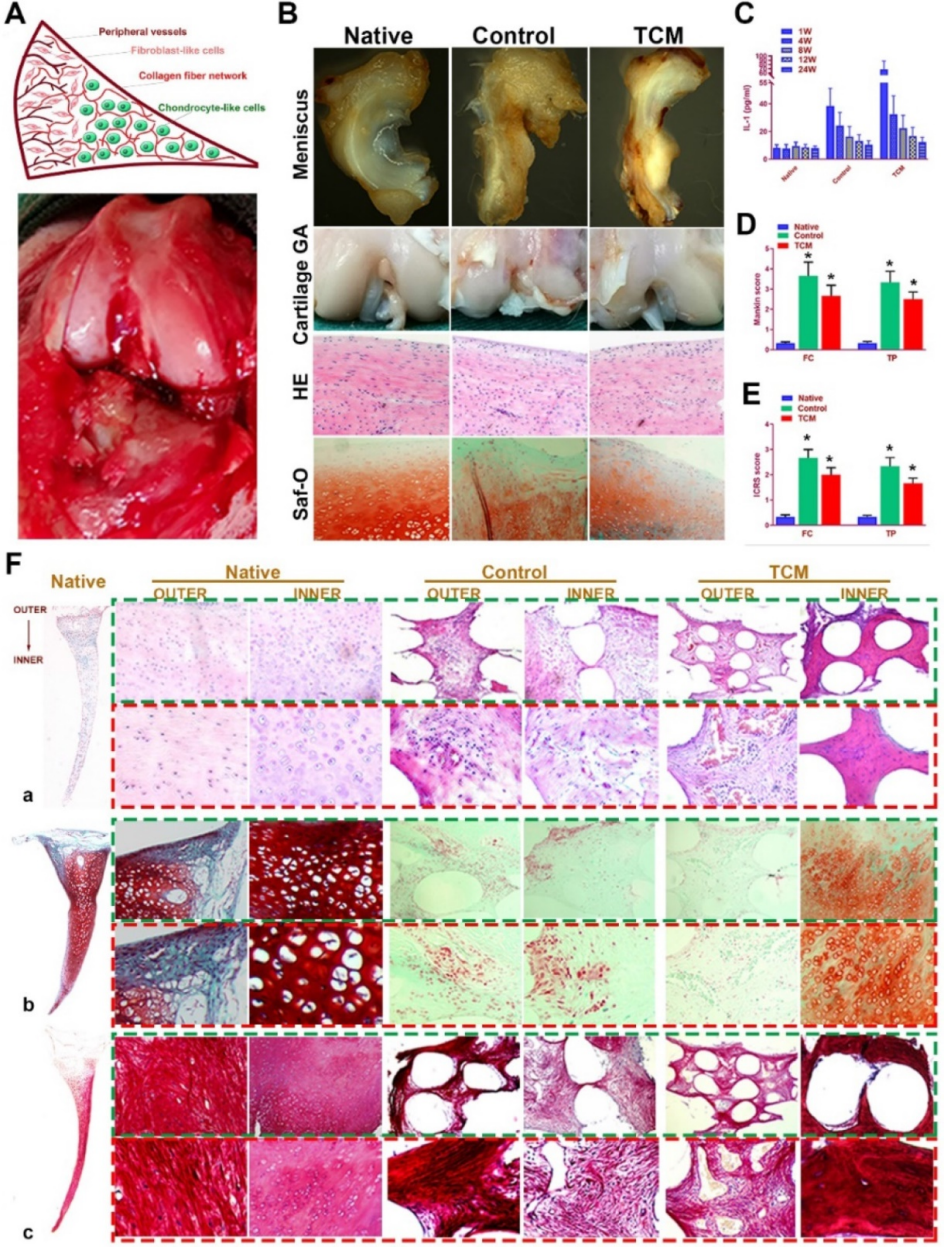
** Regeneration of rabbit meniscus resembling the native meniscus with TCM scaffold 24 weeks after transplantation* in vivo*. A.** Schematic illustration of native meniscus and the gross view of surgical procedure. **B.** Gross appearance of regenerated menisci (1^st^ row) and joint surface cartilage (2^nd^ row) at 24 weeks. Cartilage surface of femoral condyle was observed with H&E (3^rd^ row) and Safranin-O (4^th^ row) staining. **C-E.** Intra-articular IL-1 concentration (n=6 for each) post-transplantation and D) Mankin scores and E) ICRS scores to assess cartilage degeneration in femoral condyle (FC: left panels) and tibial plateau (TP: right panels). All data are means ± SD (*n* = 6) and were analyzed by two-way ANOVA with Tukey's test. *P< 0.05 between the control or TCM group and native group. **F.** Region-specific matrix phenotype analysis in generated meniscus versus native meniscus. Tissue sections of native, control and TCM meniscus were stained by **a)** HE staining (1^st^ and 2^nd^ rows) for cell phenotypes and tissue integrity, **b)** safranin-O (3^rd^ and 4^th^ rows) for proteoglycans and **c)** picrosirius red (PR) for COL-1 and COL-3 (5^th^ and 6^th^ rows). Section staining of native meniscus were listed in the left. Magnified images were listed as the lower panel for each staining.

TCM meniscus scaffold was transplanted into rabbit knee joints *in vivo* to evaluate its efficacy in joint protection and restoring the phenotype anisotropy of healthy meniscus. Total medial meniscus was dissected as previously described and the engineered meniscus construct was transplanted *in situ* (Figure 8A). At 24 weeks, rabbits were sacrificed and whole knee joints with regenerated menisci tissues were collected for observation, histological analysis and biomechanical analysis (Figure 8B; Figure S1). At 24 weeks, regenerated meniscus in the control group showed shape deformation while TCM meniscus scaffold sustained the normal meniscal gross appearance with significant greater blood vessel infiltration observed for the outer region, resembling the PBV growth in the native healthy meniscus (Figure 8B). TCM meniscus showed greater tensile modulus, aggregate modulus, higher ultimate tensile strength (UTS), and radial strength than the control (Figure S1A-C), approaching the values of healthy native meniscus (Figure 4A-H). To verify the biomechanical anisotropy the generated meniscus constructs, we conducted bidirectional tensile testing as well as compressive testing from different anatomic locations of the generated meniscus constructs (Figure S1D-H). Maximum pull-out strength of the construct-to-outer rim of the native and TCM meniscus was significantly greater than the control, indicating significantly better integration with peripheral joint capsules for the TCM meniscus (Figure S1E). At 24 weeks, both the TCM and native meniscus showed regional variations (outer versus inner) in circumferential tensile modulus, reduced modulus, and hardness. Moreover, significantly greater values in bidirectional tensile and compressive testing were found for TCM and native meniscus compared to the control (Figure S1E-H). All these results clearly indicated that TCM meniscus scaffold restored biomechanical properties and functional anisotropy resembling the healthy native meniscus at 24 weeks* in vivo*.

**Figure 9:**
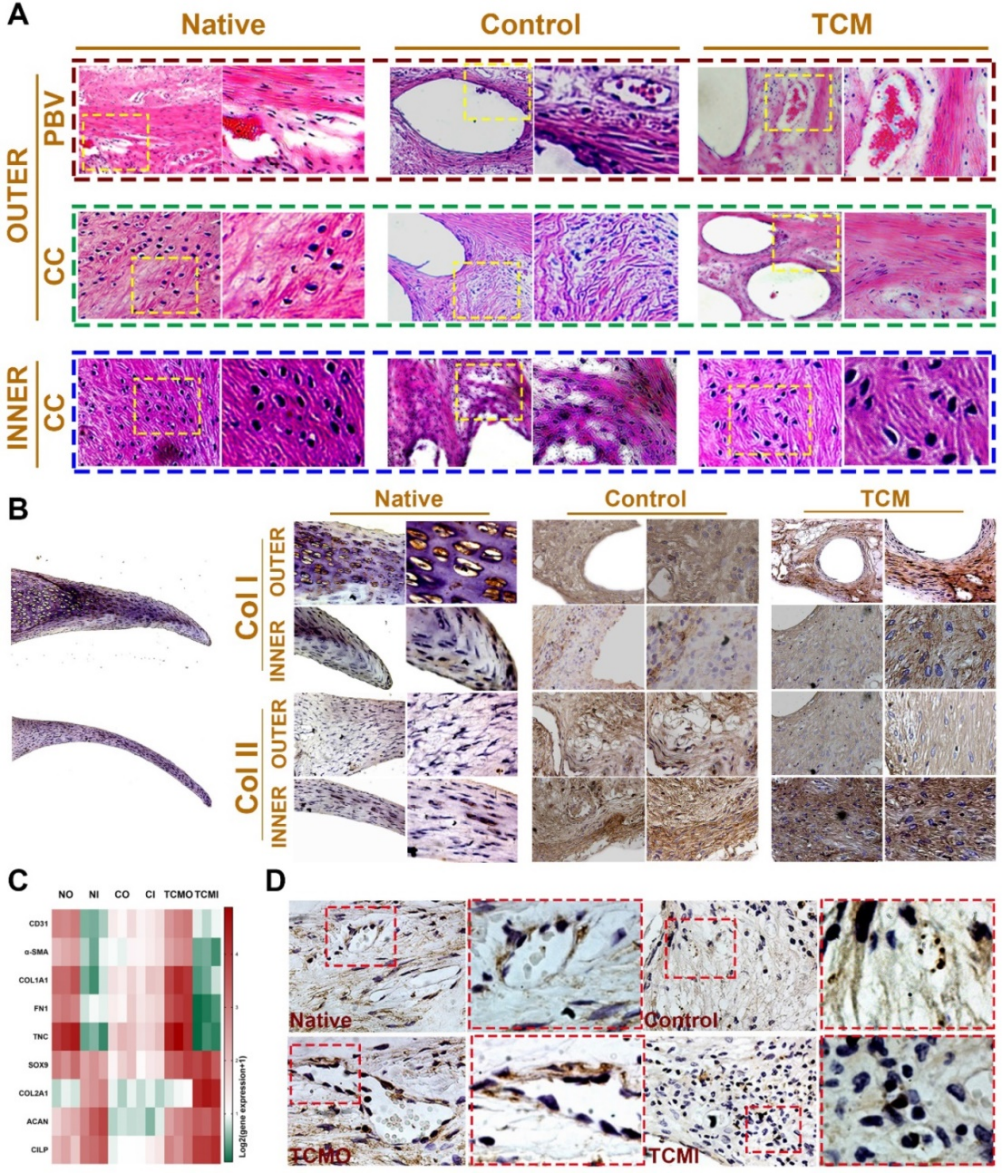
** Histological assessment of regional PBV ingrowth and cellular phenotype anisotropy in generated TCM meniscus. A.** Histological evaluation with HE staining of regional PBV ingrowth and cellular phenotype anisotropy resembling those in the native healthy tissue. Magnified images were listed as the right panel for each in the yellow box. **B.** Region-specific chondrocyte phenotype analysis in generated meniscus versus native meniscus. Inner and outer tissue sections of native, control and TCM meniscus were stained with COL I and COL II antibodies. Section staining of native meniscus were listed in the left. Magnified images were listed as the right panel for each staining. **A)** Aligned fibrous matrix and fusiform-shaped fibroblast-like cells with significantly greater PBV ingrowth and **B)** COL1A1 expression was demonstrated in the outer zone while **A)** the inner zone exhibited a cartilaginous matrix with abundant round-shaped chondrocyte-like cells with **B)** higher COL2A1 expression. **C.** Gene expression analysis of blood vessel development, articular cartilage and fibro-cartilage markers to validate the heterogeneity of phenotypes in the TCM meniscus. **D.** Immunostaining of angiogenic factor VEGF in the native and generated menisci. Similar to native meniscus, TCM meniscus demonstrated better blood vessel formation by PBV infiltration in the outer region (TCMO) than the control meniscus or the inner TCM region (TCMI). Magnified images were listed as the right panel for each in the red box.

Moreover, evaluation of joint surface demonstrated smooth cartilage surface in the TCM group and severe cartilage damage in the control group respectively 24. Greater intensity of Safranin-O staining was also observed for the TCM group compared to the control, indicating better chondroprotection of the regenerated TCM meniscus (Figure 8B). Meniscus scaffold implantation in both surgical groups caused a slight inflammatory response in the acute period. Concentration of Interleukin-1 (IL-1) and Tumor necrosis factor-α (TNF-α) in the surgical groups decreased at one month and sustained at the low level later (Figure 8C). Histological analysis of the operated joints indicated a better chondroprotective effect in the TCM group compared to the control over 24 weeks (Figure 8D-E). Joint cartilage in both control and gel groups demonstrated declined ICRS scores and greater Mankin scores compared to the native group (Figure 8D-E). TCM meniscus scaffold transplantation showed better chondroprotection with significantly higher ICRS and lower Mankin scores in the femoral condyle (FC) and tibial plateau (TP) over 24 weeks* in vivo* (Figure 8D-E). Histological evaluation revealed region-specific anisotropic phenotypes in the regenerated TCM meniscus resembling those in the native healthy meniscal tissues (Figure 8F; Figure 9). In the TCM group, Aligned fibrous ECM and fusiform-shaped fibroblast-like chondrocytes with significantly greater PBV ingrowth and COL1A1 expression was identified in the outer region while the inner region exhibited a cartilaginous ECM with numerous round-shaped chondrocytes with higher deposited proteoglycans and COL2A1 expression (Figure 8F; Figure 9A-B), suggesting the formation of anisotropic tissues resembling the healthy meniscus. Gene expression assay of blood vessel development, articular cartilage and fibro-cartilage markers further validated the heterogeneity of region-specific phenotypes in the TCM meniscus (Figure 9C-D). In summary, these results indicated that, TCM meniscus scaffold not only restored the anisotropy of native healthy meniscus with PBV infiltration and better shape retention, but better maintained rabbit knee joint function after transplantation.

In the present study, we have one-step generated 3D-bioprinted anisotropic meniscus with PBV growth and regional differential cell and ECM depositions. Transplantation of the ready-to-implant 3D-bioprinted meniscus into rabbit knees recapitulated phenotypes of native healthy meniscus and conferred long-term protection against secondary joint degeneration. Healthy meniscus was characterized by a complex anisotropic phenotype concerning blood vessel development and regional differential chondrocyte phenotypes, which was disrupted in OA development and contributed to OA progression. CTGF and Mg^2+^ were applied in the outer region to facilitate PBV ingrowth at the peripheral region and FC differentiation within type I collagen-rich ECM, contributing to its resistance against tensile loads. Vascularization with blood vessel growth within was pivotal for graft survival after transplantation. Mg^2+^ promoted HUVEC migration for tube formation and further angiogenesis in the peripheral region of the TCM meniscus, facilitating vascularization of the TCM meniscus and its integration with peripheral joint capsule tissues. SMSC-laden bioink allows sustained release of SMSC and protein-encapsulated microspheres, maintaining cell viability and promoting cellular differentiation in the TCM scaffold 25. Moreover, the supporting structure by PCL scaffolding structure offers whole-scaffold structural integrity its mechanical support, providing a stable microenvironment for those 3D-anchored SMSCs within to form meniscal tissues with secreted cartilaginous ECM. Long-term structural stability of engineered TCM construct could also be provided by PCL's long degradation time (~1 to 2 years) 26. Regionally specific ECM compositions of the one-step bio-printed TCM meniscus shared many characteristics of native healthy menisci, including PBV ingrowth, anisotropic regional expression of collagen I and II and therewith the conferred anisotropic zone-specific function and biomechanical properties. In summary, TCM meniscus not only restored the anisotropy of native healthy meniscus with PBV infiltration and better shape retention, but better maintained joint function and prevented secondary joint degeneration. However, the longer-term observation was still needed to assess whether anisotropic phenotypes of the TCM meniscus could be maintained in daily function. Taking into account the personalized design advantages of 3D-bioprinting and the individual difference of meniscus in different populations, the use of 3D-bioprinting also offered the possibility and new strategy of manufacturing “custom made” TCM meniscus for the clinical treatment of meniscus injury-related joint degenerative diseases. We envision a 3D-bioprinted anisotropic meniscus scaffold ready to implant in an arthroscopic surgery to replace the damaged or degenerated meniscus, which could be a good combination of surgery and 3D-bioprinting technology in the future.

## Supplementary Material

Supplementary materials and methods, figure and table.
