# 3D-bioprinting ready-to-implant anisotropic menisci recapitulate healthy meniscus phenotype and prevent secondary joint degeneration: Erratum

**DOI:** 10.7150/thno.73783

**Published:** 2022-05-28

**Authors:** Ye Sun, Yuxin Zhang, Qiang Wu, Feng Gao, Yongzhong Wei, Yimin Ma, Wenbo Jiang, Kerong Dai

**Affiliations:** 1 Department of Orthopaedics, The First Affiliated Hospital of Nanjing Medical University, Jiangsu, 210029, China.; 2 Clinical and Translational Research Center for 3D Printing Technology, Shanghai Ninth People's Hospital, Shanghai Jiao Tong University School of Medicine, Shanghai, 200011, China.; 3 Shanghai Key Laboratory of Orthopaedic Implants, Department of Orthopaedic Surgery, Shanghai Ninth People's Hospital, Shanghai Jiao Tong University School of Medicine, Shanghai, 200011, China.; 4 Department of Rehabilitation Medicine, Shanghai Ninth People's Hospital, Shanghai Jiao Tong University School of Medicine, Shanghai, 200011, China.

The authors regret the error in Fig. 7D of our original article. The representative image of fluorescent staining in the control group is incorrect. The correct fluorescent image has been provided in the revised Figure 7D below. We confirm that the change of this image does not affect the results and conclusion.

## Figures and Tables

**Figure 7D:**
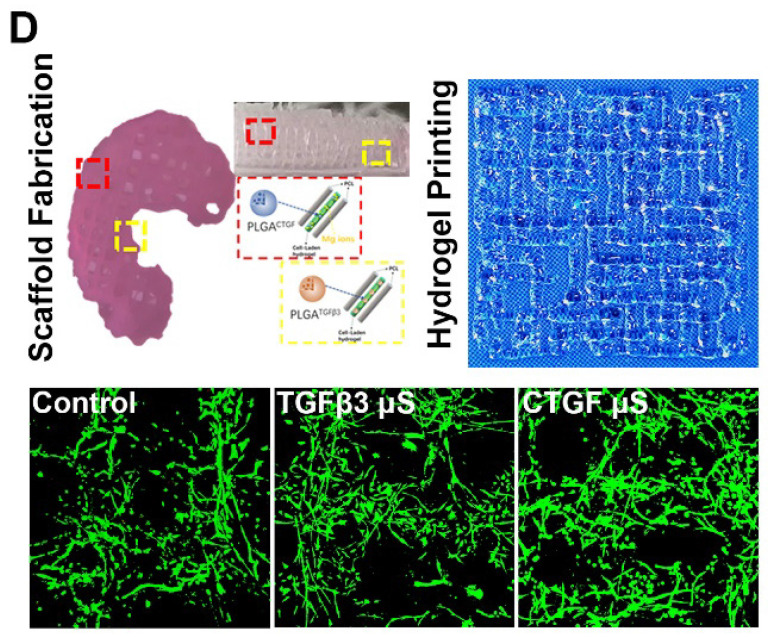
Fabricated TCM meniscus (top left) was incubated *in vitro* for 12 weeks. Addition of PLGA μS did not alter the printability of the composite hydrogel and its orderly alignment in printing (top right). Cellular interaction and viability (lower panel) was observed under microscope for different μS-conjugated hydrogel compared to that in the control group.

