# Supplementary material for: 3D-bioprinting ready-to-implant anisotropic menisci recapitulate healthy meniscus phenotype and prevent secondary joint degeneration
Source: Theranostics. 2021 Mar 5;11(11):5160–73. doi: 10.7150/thno.54864 (PMC8039947; doi:10.7150/thno.54864)
Supplement: Supplementary file 1 — Supplementary materials and methods, figure and table. [file thnov11p5160s1.pdf]

Supporting Information

3D-bioprinting ready-to-implant anisotropic menisci recapitulate healthy meniscus phenotype and prevent secondary joint degeneration

Ye Sun[1,2,3,*,#], Yuxin Zhang[4,#], ,Qiang Wu[2,#], Feng Gao[1,#],

Yongzhong Wei[1,#], Yimin Ma[1], Wenbo Jiang[2] ,Kerong Dai[2,3]

Materials and Methods

In vitro experiments

SMSCs were isolated from synovial biopsies of healthy rabbit knee joints. The SMSC pools were expanded until passage 3 at 37 °C, 5% CO2, and 95% humidity in Dulbecco's modified Eagle medium (DMEM, Life Technologies, China) with 10% fetal bovine serum (HyClone FBS, Thermo Scientific, USA), 1% antibiotic– antimycotic (100 units mL^{-1} penicillin, 100 mg mL^{-1} streptomycin, and 0.25 mg mL^{-1} amphotericin B), and 1 × 10^{-3} M sodium pyruvate (Life Technologies, China). Medium was changed every 2–3 days, and SMSCs were harvested with Trypsin (Life Technologies, China) at a confluence of 80–90%. Trypsin was used for all passaging and harvesting steps during cell handling. The ethical committee for shanghai ninth hospital (school of medicine, Shanghai jiaotong

university) approved all procedures. SMSCs were used at P2 for the following experiments. Immunofluorescence staining of fibrogenic and chondrogenic markers (Col1A1, Col2A1) was conducted to compare the TGFβ3 or CTGF microsphere-generated chondrocyte phenotype in different regions of the meniscus after 12-week incubation and observed under confocal microscopy (Leica, Japan). The expression of chondrogenesis marker (SOX9, Col1A1, Col2A1) in the meniscus constructs were analyzed by real-time polymerase chain reaction (RT-PCR) using an ABI 7300 RT-PCR system (Applied Biosystems, USA). Four-week-old tissue generated *in vitro* were stained with toluidine blue for proteoglycan production and picrosirius red for Collagen I and III. The stained images were taken using a light microscope (Leica Microsystems, Germany).

Fabrication of scaffolds

Bioprinting rabbit-derived SMSC-laden hydrogels together with biodegradable polymers was conducted for TCM meniscus construction using OPUS system (*Novaprint*, China). The MSCs suspension (a total of 1 × 10^7 cells) was loaded into the composite hydrogel (Table S1). Inspired by the heterogeneity of native meniscus structure, we produced the 3D meniscus structures by

placing together cell-laden hydrogel and PCL (~100 μm diameter for hydrogel and ~200 μm diameter for PCL) to construct a composite meniscus scaffold. Briefly, PCL was molten to fabricate the supporting structure for the scaffold while MSC cell-laden hydrogel encapsulating PLGA microparticles carrying TGFβ3 or CTGF & Mg2+ in different regions was bio-printed into the microchannels between PCL fibers from different syringes (Movie S1). During plotting, the needle diameter, layer thickness and speed for PCL printing were kept constant at 200 μm, 200 μm and 180 mm/min, respectively. The fiber spacing was kept constant at 300 μm throughout the scaffolds.

rhTGFβ3 and rhCTGF were microencapsulated in PLGA (50:50 PLA/PGA) microspheres (μS) to deliver TGFβ3 (20 ng/ml; PeproTech) and CTGF (200 ng/ml; PeproTech) & Mg^{2+} in hydrogel as previously described [1]. TGFβ3 and CTGF μS, with empty μS served as control, were mixed in the cell-laden hydrogel respectively (Table S1) and printed into the microchannels between PCL fibers with different syringes. To chemically simulate the anisotropic phenotypes in native meniscus, $PLGA^{CTGF}$ encapsulated MSC cell-laden hydrogel was used in the outer 1/3 regions while $PLGA^{TGF\beta3}$ was used for inner 2/3 regions of the

meniscus construct. SEM images of PLGA μS were taken, showing the diameter for generated PLGA μS. The printability of the PLGA encapsulated MSC cell-laden hydrogel was also shown with a test run. Release kinetics of TGFβ3 and CTGF from PLGA μS were measured by incubating microspheres (10 mg/ml) encapsulating TGFβ3 (0.1% bovine serum albumin) or CTGF (in phosphate-buffered saline) at 37 °C with mild agitation for up to 60 days. Upon centrifugation at 2500 rpm for 5 min, supernatant of the PLGA μS incubation solution was collected. Released TGFβ3 and CTGF concentration was measured using enzyme-linked immunosorbent assay (ELISA) kits following the manufacturer's protocols. To validate μS distribution in MSC cell-laden hydrogel, fluorophore-conjugated rhodamine was encapsulated in to PLGA μS and delivered to the hydrogel. At day 7, PLGA rhodamine μS distribution as well as its cell toxicity in the hydrogel was observed under confocal microscope. After 21 days of culture with chondrogenic supplemented medium, the scaffolds were harvested and analyzed for cell-anchoring and zone-specific tissue formation. Cell anchoring was confirmed by DAPI/Phalloidin staining in the scaffolds with immunofluorescence assay. Briefly, phalloidin (Thermo Fisher Scientific, USA) was used to stain the F-actin for 1 hours and incubated with DAPI (Thermo Fisher Scientific, USA) to

stain the nucleus for 5 min in turn. Cell proliferation through 21 days in the constructs was assessed with Alamar Blue assay kit (DAL1100; Life Technologies) according to the manufacturer's instruction as previously described [2].

Migration assay of HUVECs with different treatments *in vitro*

Transwell assay: A transwell culture chamber with a polycarbonate membrane (CoStar, Cambridge, MA), was used to monitor HUVECs migration in different treatment groups. Briefly, HUVECs were suspended in α-MEM with 0.2% BSA and 0.5% FCS at a concentration of 2×10^5 cells/ml. 0.1 ml aliquots of cell suspension were added to the top chamber in 24 well plate. The lower chamber contained 0.6 ml of α-MEM with 0.5% FCS and 0.2% BSA. After the cell seeded, Chemical stimuli (control, 2.5mM Mg^{2+}, 5mM Mg^{2+}, 10mM Mg^{2+} with or without CTGF) were added in the lower chamber incubation was performed for 24 hours at 37 °C. After 24 hours, cells were fixed with cold methanol after washing with PBS. The upper side was scraped gently to remove the non-motile cells. The cells were staining with crystal violet stain, and examined under a light microscope. The number of migrated cells was scored for six independent fields per transwell filter. The average was used as the cell migration efficiency.

HUVEC scratch assay in hydrogel: In order to check the wound healing potential of magnesium, HUVECs were seeded into 24-well plates pretreated with our composite hydrogel and grown to confluency. After 24 hours of serum starvation (1% FBS), lesions were made in the monolayer using cell scraper. Cells were rinsed with PBS, stained with calcein dye and then incubated in different experimental groups (control and different concentration of magnesium) for 24 hours. Cells were fixed with 4% paraformaldehyde after 24 hours and the number of cells which had moved across the starting scratched lines was measured for different groups.

Tube formation assay: The 96-well plates were pre-coated with matrigel at 37 ℃ for 2 hours and 100 μL of HUVEC suspension was added to each well at a density of 1×10^5 mL^{-l}. Then, 2.5 mM-10 mM magnesium ions, PBS, were added to the wells. After 6 hours of incubation, the tube-like structures were quantified by counting the formed tube length and the number of intersections between the branches of the endothelial cell networks in each field.

HUVEC migration in the bioprinted hydrogel and scaffolds: To demonstrate the potential of different combination of stimuli for HUVEC migration, HUVEC migration assay in the bioprinted hydrogel was conducted. HUVECs were seeded into the bioprinted

composite hydrogel, and a microchamber was punched out with a needle in the hydrogel with different stimuli combinations. After 24 hours, cells in the microchamber were rinsed with PBS, stained with calcein dye and observed with confocal microscope. The percent of recovered areas by the migrated HUVECs within was measured for different groups. To test HUVEC migration in the bioprinted scaffold, the PCL scaffolds were placed atop the monolayer-cultured HUVEC and incubated in different groups for 2 weeks *in vitro*. After 2 weeks, the scaffolds were fixed with 4% paraformaldehyde after 24 hours and cut in half. Migrated cells in the scaffolds were confirmed by DAPI staining. Briefly, scaffolds were incubated with DAPI (Thermo Fisher Scientific, USA) to stain the nucleus for 15 min. The migration distance was measured for HUVECs in the scaffolds for different groups on confocal microscope.

Animal experiment

Before meniscus replacement surgery, the scaffolds were transplanted subcutaneously for ectopic chondrogenesis in nude mice for 3 months to determine the chondrogenic properties of the 3D-bioprinted TCM meniscus scaffold. TCM meniscus was used in the TCM group while MSC-laden hydrogel/PCL composite scaffold

without TGFβ3, CTGF or Magnesium ions was used in the control group. Then, a total of 12 adult rabbits were randomly allocated into TCM (n= 6) and control (n= 6) groups for *in vivo* study. Both knees were used for experiment. Histological examination was conducted for scaffold in left knees and right knees were chosen for biomechanical analysis of the scaffolds. Briefly, a medial parapatellar approach was made through the skin and subcutaneous tissue and the patella was luxated medially. After flexing of the joint and exposure of the medial meniscus, total meniscectomy was performed using an electric scalpel and immediately grafted with 3D-bioprinted meniscus scaffolds, followed by suturing (2-0 Ethibond Excel, Braided). All the associated joint ligaments were preserved. The joint capsule and subcutaneous tissue were closed separately in continuous absorbable suture. The skin was staple-closed and covered with a sterile bandage. After the operation, rabbits were allowed to move freely in their single cages and fed with standard food and water. Twenty-four weeks later, rabbits were sacrificed for further study. The protocol was approved by the local Institutional Animal Care and Use Committee (IACUC) and complied with the Guide for the Care and Use of Laboratory Animals published by the National Academy Press (National Institutes of Health Publication No. 85-23,

revised 1996).

Assessment of regenerated meniscus and its chondroprotective effects

Serial sections (4-μm thick) were cut sagittally through the center of the generated meniscus comparing to native meniscus and stained with H&E, toluidine blue, and picrosirius red according to standard protocols. Immunohistochemical staining of markers (Collagen II and I) for chondrocyte phenotype was conducted according to standard protocols in the generated meniscus tissue sections in different groups compared to the native meniscus. The stained images were taken using a light microscope. The cartilages of the medial FC and TP were grossly evaluated according to the criteria of the ICRS cartilage lesion classification system [3]. For histological evaluation, the osteochondral specimens were sectioned on the sagittal plane at the midpoint of the medial FC and stained with H&E and Safranin-O and graded according to the Mankin grading system.

Biomechanical analysis

The biomechanical properties of the explanted scaffold *in vitro* were assessed using a material-testing machine (Instron 5843, USA).

Pull-out strength for each scaffold-to-native suture rim was calculated and compared. Bidirectional tensile testing was performed with uniaxial tests in the radial and circumferential directions within the samples. In addition, each sample (1-mm thick) was cut into a rectangular shape in the designated regions. Uniaxial tensile testing was performed on the samples in the circumferential direction as previously described [4]. The samples were tested to failure at a rate of 0.06 mm/s. The elastic modulus was analyzed from the linear portion of the stress-strain curve. In confined compressive testing, a cylindrical sample of 2-mm diameter and 1-mm thickness from the anterior section of the scaffold was used. The creep response of the specimen under a step force (0.02 N) was monitored until reaching equilibrium (defined as slope $< 1 \times 10^{-6}$ mm/s, for at least 7200 s). The test force was then automatically removed, and the recovery phase was monitored until reaching equilibrium (defined as slope $< 1 \times 10^{-6}$ mm/s, for at least 3600 s). The aggregate modulus was then calculated using the Mow biphasic theory [5]. Compressive testing in the different anatomic locations (inner or outer regions) in the tissue-engineered meniscus was also performed by nanoindentation. Briefly, samples were isolated from the inner or outer regions of the meniscus tissue *in vitro*. All indentations were

analyzed using a TriboIndenter (Hysitron Inc.) with a 400-mm radius curvature conospherical diamond probe tip. A trapezoidal load function was applied to each indent site (inner or outer region) with loading (10 s), hold (2 s), and unloading (10 s). The operation of the indentations was force-controlled to a maximum indentation depth of 500 nm. The values of reduced modulus and hardness were then calculated.

Statistical analysis

Sample sizes for all quantitative data were determined by power analysis with one-way ANOVA or two-way ANOVA. All statistical data were expressed as means ± SD. Comparisons of differences between construct regions for each stimulus group were used to test the anisotropic properties of the engineered meniscus tissue. The efficacy of the growth factors in building functional properties within different regions was also tested. One-way ANOVA or two-way ANOVA with Tukey's test was used to analyze the data. When the two-way ANOVA showed significance ($P < 0.05$), Tukey's test was applied. Interaction effects were estimated using a general linear model. All data analyses were performed using SPSS statistical software (version 15.0; SPSS Inc.). Values of $P < 0.05$ were considered statistically significant.

Reference

1. Shi D, Xu X, Ye Y, Song K, Cheng Y, Di J, et al. Photo-cross-linked scaffold with kartogenin-encapsulated nanoparticles for cartilage regeneration. ACS Nano. 2016; 10(1): 1292-9.
2. Kang HW, Lee SJ, Ko IK, Kengla C, Yoo JJ, Atala A. A 3D bioprinting system to produce human-scale tissue constructs with structural integrity. Nat Biotechnol. 2016; 34(3): 312-9.
3. Brittberg M, Winalski CS. Evaluation of cartilage injuries and repair. J Bone Joint Surg Am. 2003; 85-A Suppl 2: 58-69.
4. Patel JM, Merriam AR, Culp BM, Gatt CJ Jr, Dunn MG. One-year outcomes of total meniscus reconstruction using a novel fiber-reinforced scaffold in an ovine model. Am J Sports Med. 2016; 44(4): 898-907.
5. Mow VC, Kuei SC, Lai WM, Armstrong CG. Biphasic creep and stress relaxation of articular cartilage in compression? Theory and experiments. J Biomech Eng. 1980; 102: 73-84.

Table S1. Preparation of the cell-laden composite hydrogels for 3D-bioprinted meniscus constructs

Gelatin	Fibrinogen	HA	Glycerol	Cell type & density
45 mg/ml	30 mg/ml	3 mg/ml	10% v/v	SMSCs, 1×10^7 cells/ml

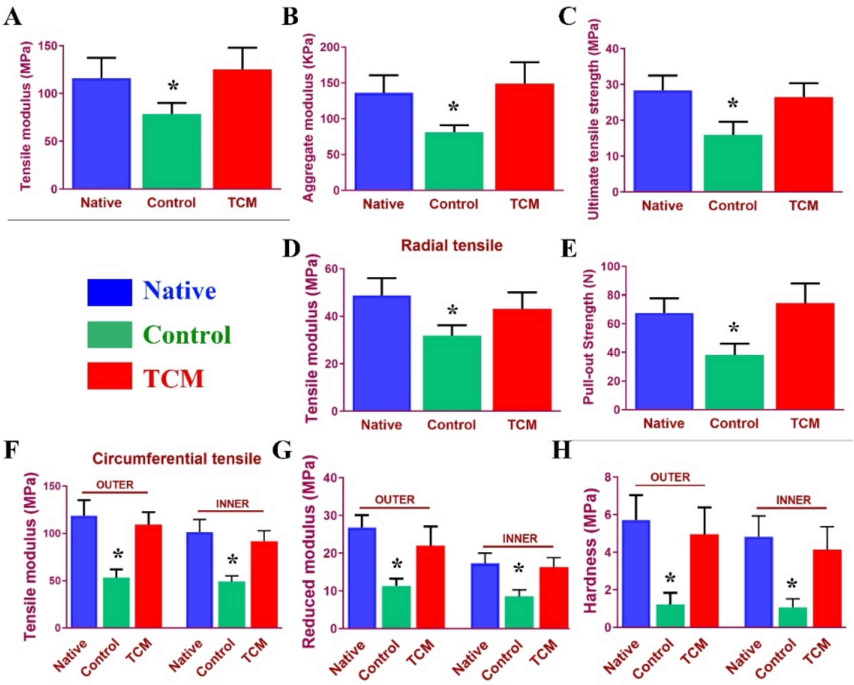

Figure S1. Biochemical properties of the regenerated knee meniscus explant after 24 weeks *in vivo*. A-D. Biomechanical properties of the *in vitro* construct, including bulk tensile modulus, aggregate modulus, and UTS after 24 weeks *in vivo*. All data are means ± SD (n = 6) and were analyzed by two-way ANOVA with Tukey's test. **D-G.** Biomechanical properties for the TCM group in bidirectional tensile testing and compressive testing in different locations on the explanted meniscus tissue constructs at 24 weeks. **D)** Radial tensile modulus. All data are means ± SD (n = 6) and were analyzed by one-way ANOVA with Tukey's test. **F)**

Circumferential tensile modulus. All data are means ± SD (n = 6) and were analyzed by two-way ANOVA with Tukey's test. **G-H.** Reduced modulus **G)** and hardness **H)**. All data are means ± SD (n = 6), and were analyzed by two-way ANOVA with Tukey's test. Data are presented as averages ± SD and were analyzed by two-way analysis of variance (ANOVA) with Tukey's test. *P< 0.05 between control or TCM group and the native group.
